# Potential influence of antimicrobial resistance gene content in probiotic bacteria on the gut resistome ecosystems

**DOI:** 10.3389/fnut.2023.1054555

**Published:** 2023-02-01

**Authors:** Marina Radovanovic, Dusan Kekic, Ina Gajic, Jovana Kabic, Milos Jovicevic, Natalija Kekic, Natasa Opavski, Lazar Ranin

**Affiliations:** ^1^Department of Biochemistry, Institute for Biological Research “Siniša Stanković”, National Institute of Republic of Serbia, University of Belgrade, Belgrade, Serbia; ^2^Institute of Microbiology and Immunology, Medical Faculty, University of Belgrade, Belgrade, Serbia; ^3^Clinic for Infectious and Tropical Diseases, University Clinical Centre of Serbia, Belgrade, Serbia

**Keywords:** antimicrobial resistance (AMR) genes, gut microbiome, gut resistome, probiotic bacteria, mobile genetic elements (MGEs)

## Abstract

Antimicrobial resistance (AMR) poses a substantial threat to human health. The commensal bacteria of the gut microbiome were shown to serve as a reservoir of antibiotic resistance genes (ARGs), termed the gut resistome, which has the potential to transfer horizontally to pathogens and contribute to the emergence of drug-resistant bacteria. Namely, AMR traits are generally linked with mobile genetic elements (MGEs), which apart from disseminating vertically to the progeny, may cross horizontally to the distantly related microbial species. On the other hand, while probiotics are generally considered beneficiary to human health, and are therefore widely consumed in recent years most commonly in conjunction with antibiotics, the complexities and extent of their impact on the gut microbiome and resistome have not been elucidated. By reviewing the latest studies on ARG containing commercial probiotic products and common probiotic supplement species with their actual effects on the human gut resistome, this study aims to demonstrate that their contribution to the spread of ARGs along the GI tract merits additional attention, but also indicates the changes in sampling and profiling of the gut microbiome which may allow for the more comprehensive studying of the effects of probiotics in this part of the resistome.

## Introduction

Antimicrobial resistance (AMR) has developed into a substantial and acute global health threat, leading to increasing death tolls worldwide (1). Thus, it has become a focus of recent public healthcare interventions and research activities. From the point of view of AMR research advancement, an important stepping stone was the introduction of the concept of resistome. This term refers to the set of resistance determinants in various contexts (a genome, a community or population, an ecosystem, and so on up to the biosphere as a whole), within which AMR determinants can evolve, before potentially crossing to human pathogens and contributing to the emergence of drug-resistant bacteria (2). This has led to the understanding that human bodies with the multiple ecosystems they contain, may also represent the vessels of AMR development and dissemination (3). One such reservoir of all circulating antibiotic resistance genes (ARGs) and their combined abundances is present in the human gut microbiome and is termed the gut resistome (4). Bacterial ARGs are generally mostly linked to various mobile genetic elements (MGEs) and thus may use the means of horizontal gene transfer (HGT) to cross into variously related species, including pathogenic (5, 6). Because of their specificities, the frequency of these ARG transfer events depends on a multitude of factors, including the taxonomic and ecological similarity between donors and recipients, the classes of associated MGE carriers, the distribution and abundances of ARGs within commensal microbes, as well as the environmental conditions and stressors in the microbial niche (4). Upon acquiring ARGs and gaining the ability to metabolize the antimicrobials and modify the levels of their influx, efflux and access to target sites, commensals, symbionts and opportunistic microbes of the human gut diminish antimicrobial access to their essential cellular functions and may increase fitness and survival compared to other bacteria in the presence of many xenobiotics or antibiotics in the gut ecosystem, including β-lactams, aminoglycosides, macrolides, tetracyclines, vancomycin, fluoroquinolones, and several other antibiotics (5, 6). Additionally, MGEs that resistance traits are often linked with, encode for different mobility functions and DNA recombinases for efficient chromosomal DNA integration of recipient bacteria, which may ultimately provide passive replication of the traits and their vertical transfer to the progeny (7–9). Thus, antibiotics represent a significant contributor to gut resistome expansion and evolution (10, 11). Recent results reflect the possibility of ARG transfer from gut commensals to pathogens, as well as the transfer of ARGs from pathogens to commensals in patients, as well as through the food chain (5, 6).

On the other hand, probiotics are defined as live microorganisms with health benefits when consumed in adequate amounts (12). At present, probiotics are one of the most commonly consumed food supplements worldwide, constituting a constantly growing market, expected to reach 91 billion dollars in 2026 (13). Probiotics have been commercialized and consumed in multiple forms: in fermented or enriched drinks and foods, including yogurts, cheeses, nutrition bars, cereals and even infant formulas, as well as lyophilized pills (14). This has been widely supported by the medical community, with many studies implying that various diseases could be treated or prevented by probiotic-induced regeneration of the gut microbiota balance, although the efficacy of probiotics as prophylactic and treatment avenues remains conflicting and debated (15–19). Nevertheless, with the context of this review in mind, the most significant is the fact that because of the widely accepted notion that they are beneficiary in reducing antibiotic-induced dysbiosis and gut resistome expansion, probiotics have most commonly been consumed in conjunction with antibiotics, although the complexities and extent of their modulating impact on the gut microbiome and resistome have not been fully elucidated (6, 20, 21).

## Aims and methods

This review is based on the results of the most recent studies demonstrating the presence of ARGs in commercial fermented drinks and food-related products, as well as in the genomes of common probiotic supplement species and strains. These data are linked with the studies that regard the gut microbiome and resistome as ecological niches involving complex interactions with numerous factors, including antibiotics and probiotics as evolving organisms, to highlight that the selection and transferability of probiotic ARGs to commensals and pathogens of the gut resistome may change depending on the environmental context. Thereby, the review aims to show that the influence of probiotics on the gut resistome merits more detailed attention, additionally pointing to a number of changes in sampling and profiling of the gut microbiome which could allow for more realistic and in-depth future studies of the contribution of probiotics to the spread of ARGs along the human GI tract.

To achieve the aforementioned goals, the National Library of Medicine (PubMed) research of relevant publications was limited to the last 5 years only, as well as by using the key terms: (“probiotic bacteria”) OR (“antimicrobial resistance genes”) OR (“gut resistome”). The overview was further progressed by analyzing studies containing genetic and/or genomic analyses of the ARG content and ARG mobility of commercial and medicinal probiotic products, wherever possible including the details regarding the species and strains. Particular attention was paid to the studies where this was combined with evidence of the specificities of ARG evolution and ARG effects on gut resistome ecosystems in various conditions, with a particular focus on the conditions created by antibiotic treatment.

## ARGs in fermented foods and beverages

As mentioned above, one of the most prominent ways probiotic bacteria can reach the human gut is through the intake of popular fermented products. In both cases, production requires milk fermentation, in the course of which the fermenting probiotic bacteria multiply and come to dominate the beverages. Consequently, the amount of potentially present ARGs in seed bacterial cultures and milk itself greatly increases in the final products (22). After ingestion, ample and diverse bacterial strains of these foods and their ARGs enrich the gut resistome and may come into close physical proximity to commensals and/or pathogens of the consumer. Thus, the probability of HGT in the gut is increased as well as the potential for the evolution of resistant or multi-resistant pathogenic strains. Importantly, since apart from the fermenting bacteria production of kefir requires additional fermenting fungi, which may produce antibacterial toxins, bacteria containing ARGs gain a competitive advantage in kefirs, and may be more variable in kefir than in yogurt (22).

The presence and transferability of genotypic and phenotypic AMR markers were investigated in several recently published studies of fermented milk products. One of them involved the lactic acid bacterial isolates obtained from such products in different areas of China (23). The isolates included strains of *Lactobacillus bulgaricus*, *Lacticaseibacillus casei*, *Lacticaseibacillus rhamnosus*, *Lacticaseibacillus paracasei*, *Lactobacillus acidophilus*, and *Streptococcus thermophilus* that were phenotypically tested to ampicillin, streptomycin, gentamicin, kanamycin, tetracycline, chloramphenicol, erythromycin, ciprofloxacin, clindamycin, trimethoprim, and vancomycin susceptibilities by the micro broth dilution method. High-level intrinsic resistance was revealed among the tested species, and the following genetic determinants of antibiotic resistance were identified: *tetM* in *Lactobacillus bulgaricus* and *Streptococcus thermophilus* isolates, *strA* and *strB* in *Streptococcus thermophilus*; *sul1* in *Lactobacillus bulgaricus* and *Streptococcus thermophilus*, *sul2* in *Streptococcus thermophilus*, *aac(6′)-aph(2′)* in *Lactobacillus bulgaricus*, as well as *aph(3′)-II* and *aph(3′)-III* in *Streptococcus thermophilus* and *Lactobacillus bulgaricus*. Of note, these were the first-time identifications of *strA*, *strB*, *sul1*, *sul2*, and *aph(3′)-II* in *Streptococcus thermophilus*, as well as of *sul1*, *aac(6′)*, and *aph(2′)* genes in *Lactobacillus bulgaricus* (23).

The *tetM* gene is generally located on various MGEs, including plasmids and conjugative transposons and encodes for the TetM protein which confers tetracycline resistance by ribosome binding and protection (24). Streptomycin resistance is conferred by *strA-strB* genes, encoding for streptomycin-inactivating enzymes. *StrA-strB* of human and animal resistomes are often linked with the *suI2* located on the non-conjugative plasmids. Plant resistome-derived *strA-strB* genes are located on a specific Tn3-type transposon, generally borne on conjugative plasmids. S*trA-strB* genes are widely distributed in the aforementioned resistomes, which implies that gene transfer events between human, animal and plant resistome-associated bacteria have occurred. Although the use of streptomycin in clinical medicine and animal husbandry has diminished, the persistence of *strA-strB* genes in bacterial populations suggests that factors other than direct antibiotic selection are involved in the preservation of these genes (25). Finally, sulfonamide resistance-conferring genes, s*ul1* and *sul2* encode for different forms of dihydropteroate synthase. While the *sul1* gene is normally linked to other resistance genes in class 1 integrons, *sul2* is usually located on small non-conjugative plasmids or large transmissible multiresistant plasmids (26, 27). Thus, most AMR conferring genes identified in the described study are located on MGEs, indicating potential risks of expansion of these ARGs from lactic acid bacteria to humans through fermented milk products.

In a more recent study using a unified metagenomic approach, the diversity of ARG content in various kefir and yogurt samples (products, grains, bacterial strains) was examined (22). Even with the strictest filter restrictions (only reference ARGs that fit the open reading frames (ORFs) from the starting base position were selected), numerous ARGs (which undermine the efficacy of aminocoumarins, aminoglycosides, carbapenems, cephalosporins, cephamycins, diaminopyrimidines, elfamycins, fluoroquinolones, fosfomycin, glycylcyclines, lincosamides, macrolides, monobactams, nitrofurans, nitroimidazoles, penams, phenicols, rifamycins, tetracyclines, and triclosan) were found in fermenting bacteria widely used in the production of fermented dairy products. These bacteria were identified by taxon classification of ARG-containing contigs and included *Bifidobacterium animalis*, *Enterobacter hormaechei*, *Lactobacillus acidophilus*, *Lactobacillus delbrueckii*, *Lactobacillus helveticus*, *Lactobacillus kefiranofaciens*, *Lactiplantibacillus plantarum*, *Lactococcus lactis*, and *Leuconostoc mesenteroides*. The two most abundant ARGs were *poxtA* and *aph(3′)-IIb*, presenting in both yogurt and kefir samples. Specifically, in this study, *aph(3′)-IIb* gene was linked with *Leuconostoc mesenteroides*, while the *poxtA* gene was associated with *Lactobacillus helveticus* and *Lactiplantibacillus plantarum.* Contrastingly, cell-free supernatant containing specific strains of *Lactiplantibacillus plantarum* (22F and 25F) was very recently shown to be a potentially useful antibiotic alternative showing antibiofilm and anticonjugation activity against the colistin-resistant, *mcr-1*–containing *Escherichia coli* from various livestock isolates (28). These strains could thus show useful as possible new avenues of management of colistin-resistant *E.coli* in livestock. *Lactococcus lactis* associated chromosomally located *lmrD* gene encoding for the efflux component of the ABC lincosamide efflux pump, with ORFs possibly providing its mobility, was also found in kefir samples. Macrolide resistance-conferring and transposon carried *emrB* gene was identified in a contig associated with the genome of *Enterobacter hormaechei*. Relative abundances of ARG-carrying *Lactobacillus kefiranofaciens* and *Leuconostoc mesenteroides* increased during fermentation. Suitably, ARG abundances expressed as fragments per kilobase per million fragments (FPKM), showed a positive association with the relative abundances (%) of their most probable bacteria of origin. For instance, an increase in the relative abundance of *Leuconostoc mesenteroides* was followed by the FPKM increase of *aph(3′)-IIb*, while *poxtA* FPKM dropped simultaneously with the *Lactobacillus kefiranofaciens* abundance (22).

The *aph(3′)-IIb* gene belongs to the corresponding group of genes, encoding for enzymes which modify aminoglycosides by acetylation, adenylation, or phosphorylation. Its presence in the kefir samples is particularly worrying since high-level resistance against several aminoglycosides may occur. Fortunately, *aph(3′)-IIb* is chromosomally encoded (22). On the contrary, *poxtA* is encoded on putative MGEs, which facilitates its rapid spread. The protein it encodes (*PoxtA*) is one of the ATP binding cassette (ABC) proteins of the F lineage which confer resistance to a wide range of critical antibiotics, namely, phenicols, oxazolidinones and tetracyclines, by protecting the ribosome, and changing the structure of the antibiotic binding site (29, 30). This protein was mainly characterized in *Enterococci* and *Staphylococci*. It was also identified in a methicillin-resistant *Staphylococcus aureus* (MRSA) strain that showed decreased susceptibility to oxazolidinone–linezolid. Most significantly, *poxtA*–harboring bacteria are all of animal origin which is why they can be spread horizontally (29). Therefore, several studies indicate that phenicols and oxazolidinones which are still used in veterinary medicine in many countries, while prohibited in alimentary animals and dairy cattle in the European Union, may have played a role in the selection of *poxtA* (29, 31, 32). Contradictorily, ARGs conferring resistance to β-lactams (penicillins and cephalosporins) as the most frequently used antibiotics in dairy cows, were not correspondingly abundant in the studied fermented milk products (22). Combined with the fact that ARGs related to antibiotics which are not used in dairy medicine show higher FPKMs in kefirs and yogurts (22), these findings raise suspicion that the selection of ARGs present in artisanal kefirs and yogurts is directly consequent to antibiotic exploitation at dairy farms.

Thus, the herein described findings indicate that the causes and routes of ARG selection, multiplication and spreading are highly complex and difficult to follow and that the established modalities for monitoring and control of the use of antibiotics in animal husbandry and products of animal origin, even if strictly applied, may not be sufficient to stop ARG evolution. Furthermore, according to the strict procedures and recommendations of the European Food Safety Authority–EFSA and the US food and drug administration authority (FDA), bacteria intended for human or animal nutrition, or as starter cultures during fermented foods production, undergo mandatory AMR testing and ARG content analysis (33–35). Despite these checks, foods of animal origin, including fermented foods and beverages, obviously still contain a great variety and abundance of bacterial ARGs. Some of them may have evolved in the presence of other fermenting organisms necessary for the fermenting process and increased their abundance in the course of fermentation. Therefore, one of the possibly desirable future avenues in the analysis of both natural and standardized starter cultures and final fermented products would include registering the results at set time points during the fermentation period, with sequencing depths of at least 20 million clusters recommended to secure ARG identification (22). Additional examinations of fermented foods and drinks are necessary to understand the real medical significance of these ARGs.

## AMR and associated ARGs in probiotic bacteria isolated from commercially available dietary and medicinal supplements

In a study by Selvin et al. (36) antibiotic susceptibility of samples from commercially available probiotic dietary supplements containing probiotics, specified strains of *Streptococcus faecalis* and *Bacillus mesentericus* showed resistance to penicillin G, while *Lactobacillus acidophilus* was resistant to ampicillin and all isolates were resistant to ceftazidime. Furthermore, *Lactobacillus sporogenes*, *Streptococcus faecalis*, *Bacillus mesentericus*, *Lactobacillus rhamnosus*, and *Lactobacillus sporogenes* demonstrated resistance to erythromycin. Nevertheless, the genetic determinants behind these phenotypes were not specified (36). However, in a more detailed, ARG-detection-oriented bioinformatic analysis of the freely available next-generation sequencing-acquired metagenome datasets of probiotic products (various powders, supplements, and lyophilized capsule contents), it was demonstrated that common probiotic bacterial strains designed for human consumption not only carry ARGs in significant amounts but in many cases, these ARGs may be transferable on MGEs and could contribute to the spread of decreased antibiotic susceptibility among other bacteria, thus possibly influencing the efficacy of multiple antibiotic treatments (37).

According to the mean prevalence, the dominant bacterial genera of the studied metagenomes included *Lactobacillus* (40%), *Enterococcus* (35%), *Bifidobacterium* (34%), *Limosilactobacillus* (34%), *Lactococcus* (32%), *Lacticaseibacillus* (31%), *Bacillus* (26%), *Weizmannia* (22%), *Ligilactobacillus* (19%), *Streptococcus* (18%), *Lactiplantibacillus* (12%), and *Sphingobacterium* (2%), most of which are core members of commercially available probiotics. Only *Sphingobacterium* and *Weizmannia* spp. are the less common components of probiotic products (37). While at least one ARG was found in each metagenomic sample, less than half of the isolates contained any of them. Namely, only contigs originating from *Bacillus subtilis*, *Bifidobacterium animalis*, *Bifidobacterium bifidum*, *Bifidobacterium breve*, *Bifidobacterium longum*, *Enterococcus faecalis*, *Enterococcus faecium*, *Escherichia coli*, *Lactococcus lactis*, and *Streptomyces albulus* contained ARGs. Nevertheless, more than 70 ARGs were identified in this study (37). Among them, *rpoB* mutants conferring resistance to rifampicin, mosaic tetracycline resistance gene, *tet(W/N/W*) and clinically significant extended-spectrum beta-lactamase (ESBL) encoding TEM-116 gene (confers resistance to penicillins, three generations of cephalosporins and aztreonam) were the most common (37).

Thus, it was determined that ARGs belonging to *Bacillus subtilis* may play a role in resistance against aminoglycosides, lincosamides, macrolides, oxazolidinones, phenicols, pleuromutilins, streptogramins, and tetracyclines, the ones of *Bifidobacterium animalis, Bifidobacterium longum* and *Bifidobacterium breve* may decrease susceptibility to rifamycins and tetracyclines, and of *Bifidobacterium bifidum* to mupirocin. Also, an increased resistance was shown for *Enterococcus faecalis* to acridine dye, diaminopyrimidines, fluoroquinolones, lincosamides, macrolides, oxazolidinones, phenicols, pleuromutilins, rifamycins, streptogramins, and tetracyclines; *Enterococcus faecium* to aminoglycosides, lincosamides, macrolides, oxazolidinones, phenicols, pleuromutilins, streptogramins, and tetracyclines; whereas *Escherichia coli* showed genetic potential for resistance to acridine dye, aminocoumarins, aminoglycosides, benzalkonium chlorides, carbapenems, cephalosporins, cephamycins, fluoroquinolones, fosfomycin, glycylcyclines, lincosamides, macrolides, monobactams, nitroimidazoles, nucleosides, penams, phenicols, rhodamines, rifamycins, tetracyclines, and triclosan. Genetic determinants of resistance to lincosamides were assigned to *Lactococcus lactis*, while determinants linked with *Streptomyces albulus* possibly enable resistance to aminoglycosides. Furthermore, based on the variety of all ARGs found in the studied probiotics, by far the most dominant mechanism of AMR involved antibiotic efflux, whereas antibiotic inactivation, antibiotic target alteration and protection and reduced permeability to antibiotics, together with various combinations of these mechanisms should be much less represented (37).

Importantly, based on the newly proposed distance method–MobileElementFinder, MGE-associated ARGs were detected in three species: *Bifidobacterium animalis*, *Enterococcus faecalis*, and *E. coli* (37, 38). Namely, *tet(W/N/W)* was associated with *ISBian1* transposable element on *Bifidobacterium animalis* originating contigs, while *tetM* linked to the transposon *Tn6009* was predicted on *Enterococcus faecalis* originating contigs. Multidrug resistance *mdtG* determinant and polymyxin B resistance *ugd* gene were associated with the *Escherichia coli* linked contigs on *IS3* and *IS100* insertion sequences, respectively. *E. coli* originating contigs were also associated with the *ISKpn24* element containing the multidrug resistance determinants *mdtE* and *mdtF*, as well as with the *IS102* insertion sequence linked to the multidrug resistance efflux *emrY* and *emrK*, and transcription regulating, *evgA* and *evgS* determinants. Additionally, respective contigs originating from *Bifidobacterium longum* and *Bifidobacterium animalis* were linked with plasmids containing the *tet(W/N/W)* gene. Interestingly, contigs of respective plasmids containing the aminoglycoside inactivating acetyltransferase [*AAC(6′)-Ii*] and macrolide resistance-conferring *msrC* genes were detected in the samples of *Enterococcus faecium*. Respective samples of *E. coli* contained plasmid contigs harboring *blaTEM*-116 and multidrug resistance determinants *marA* and *marR*. Finally, exclusively dsDNA phages were detected by phage prediction (37). Specifically, one contig, assigned to the metagenomic *Bacillus subtilis*–contained prophage harbored the gene *aadK*, encoding for the streptomycin-modifying aminoglycoside-6-adenylyltransferase (39). Prophages in contigs predicted to originate from metagenomic samples and isolates of *Enterococcus faecalis*, contained the antibiotic efflux-linked gene *efrA* (40). One *E. coli*–assigned contig had the gene TEM-116, while the one linked with *Lactococcus lactis* carried *lmrD* within a prophage (37). Interestingly, all *E. coli* isolates contained contigs with prophages harboring ARGs, specifically previously mentioned *marA*, *marR*, *emrK*, *emrY*, *evgA*, as well as the multidrug resistance determinant, *mdfA* (41), β-lactamase *ampC* (42) and aminoglycoside and β-lactams resistance determinant, *cpxA* (37).

Such knowledge raises important clinical considerations, being that identified ARGs, most worryingly the ones located on designated MGEs (insertion sequences, plasmids, and phages), may undermine treatments with some of the most frequently administered antibiotics worldwide. Namely, out of the antimicrobials classified as critically important for human medicine in the latest WHO report, treatments of various diseases with aminoglycosides, carbapenems, cephalosporins, glycylcyclines, macrolides, monobactams, oxazolidinones, penicillins, and quinolones could be affected by the ARGs identified in such studies, if these ARGs were transmitted to the corresponding pathogenic bacteria of the consumer (43).

Importantly, the described features of probiotic species are the results of *in silico* predictions of the locations and mobility of ARGs. Additional functional studies are still needed to determine whether the identified ARGs truly operate in the bacterial strains of given probiotics. Furthermore, constituent strains of probiotics can often be or are intentionally made multiresistant, so that they can be co-administered with oral antibiotics and reduce their gastrointestinal side effects. As briefly highlighted above, some ARGs may also play a role in the probiotic effect of the carrying bacteria and provide increased fitness against specific toxins (44, 45). Thus, it seems that certain ARGs are essential for the efficient colonization of the gut by many beneficial bacteria, but the selection of probiotic strains should be carried out with greater prudence and detailed monitoring of the MGE-located ARG content of probiotic preparations and species, in order to exclude the possibility of serious consequences on human health due to their consumption.

With this in mind, a well-designed example is a recently performed study by Pell et al., which involved the whole genome sequencing and analysis of a *Lactiplantibacillus plantarum* ATCC 202195 probiotic strain. Specifically, its principal aims were formulated with a final goal to perform the design of future studies employing this probiotic and synbiotic component, by characterizing the genetic determinants of its main phenotypes (46). Namely, oral administration of a synbiotic containing *Lactiplantibacillus plantarum ATCC 202195* and fructooligosaccharide (FOS) to newborns was previously shown to lead to sustained intestinal colonization and reduce the risk of sepsis in newborn infants (47, 48). According to the identified gene pool, the potential mechanisms by which *Lactiplantibacillus plantarum* exerts these effects include stress responses, cellular adhesion, carbohydrate metabolism, and vitamin biosynthesis. These mechanisms also support the probiotic properties of this bacterium, as well as its use as a synbiotic in combination with FOS. Furthermore, the genetic identity of the recently commercialized strain of *Lactiplantibacillus plantarum ATCC 202195* was confirmed, relative to the isolate patented 20 years prior, as well as to the one used in trials showing its clinical significance (46). However, concerns regarding its safety, mainly potential virulence and phenotypically profiled antimicrobial resistance, were also not previously addressed. Thus, the genetic characterization of potential virulence factors of *Lactiplantibacillus plantarum ATCC 202195* and its ARGs was also performed (46). Specifically, antimicrobial susceptibility to a panel of 20 clinically relevant antimicrobial agents, ranging across 16 classes of antibiotics, was corroborated with the chromosomal and plasmid sequencing data, to establish if AMR of *Lactiplantibacillus plantarum ATCC 202195* is intrinsic or has the potential to be transferred to other microorganisms. Its two respective isolates had identical *in vitro* antimicrobial susceptibility profiles, with rifampin and penicillin MICs falling within one doubling dilution of the upper range of previously reported MICs (49). Furthermore, screening the genomes of both patented isolates of *L. plantarum* ATCC 202195 for sequence homology with various ARGs, revealed only partial hits, with low sequence identity and coverage to the resistance-conferring, mutated multidrug efflux heterodimers *LmrCD* and *rpoB* (β-subunit of RNA polymerase) (49, 50). Although *in vitro* antimicrobial sensitivity assay results were indicative of rifampin resistance, a conclusive statement in this regard could not be made, since established clinical breakpoints were lacking. A partial match to a tetracycline resistance gene *tetM*, presumably responsible for the corresponding phenotypic resistance, was identified. Both isolates were resistant to vancomycin, which was corroborated by the conserved presence of the signature active site mutation in the *ddl* gene within the genomes of the patented *L. plantarum* ATCC 202195 isolates. Furthermore, high ciprofloxacin MICs were also suggestive of resistance. However, since no fluoroquinolone-resistant mutations identified the target *gyrA* gene, it was suggested that the ciprofloxacin resistance phenotype of *L. plantarum* ATCC 202195 could be attributed to its increased efflux. Finally, both isolates were sensitive to ampicillin and gentamicin (46). Since the WHO currently recommends the combination of these antibiotics for the initial empiric treatment of neonates with suspected sepsis, the aforementioned finding confirms the safety of *L. plantarum* ATCC 202195 use in the prevention of neonate infections (46, 51). Finally, in contrast to previous reports, two putative plasmids were characterized in both isolates of *L. plantarum* ATCC 202195, but no concerning plasmid-encoded ARGs were found (48, 52). Thus, it was shown that *L. plantarum* ATCC 202195 still does not pose a material threat to the physical transfer of ARGs to other microorganisms (46).

## Influence of probiotics and different assaying on the gut microbiome and resistome

The influence of probiotic bacteria on host microbiomes and resistomes, most importantly the one situated in the GI tract, may extend beyond the potential direct transfer of ARGs to commensals and pathogens. Additionally, it was recently suggested to depend on the context in which it is exerted, such as the person-specific characteristics of the gut microbiome and the presence of other agents, including antibiotics (6). Previously, the use of antibiotics was shown to contribute to gut resistome expansion (10, 11). As mentioned before, the transfer of ARGs between pathogens and commensals and vice-versa has also been demonstrated experimentally, in patients and through the food chain (6). Contrarily, probiotics have been hailed as means for restoring the gut microbiome balance and preventing gut resistome expansion, particularly after perturbation by antibiotics (21). The extent of gut microbiome modulation together with the effects of probiotics on the gut resistome is still largely unclear. Many of these unknowns may stem from the inadequate sampling of the GI microbiome. Namely, most contemporary studies exclusively rely on stool sampling, thus reflecting the GI microbiome only partially and not taking the individual specificities of the GI probiotic colonization into account (6, 53). Furthermore, in the study of Montassier et al., it was shown that the functional gene content differs between stool and endoscopy-collected GI samples not only in the whole microbiome but in the gut resistome specifically. Namely, the detected α-diversity was significantly lower in stool samples, suggesting that stool samples under-represent the GI tract resistome and that the use of endoscopic samples for proper assessment of the probiotic effects on the gut resistome is strongly preferable. Thus, the authors decided to analyze an existing metagenomic dataset to characterize the ARG content of the paired colonoscopy, performed before and during supplementation with probiotics, and stool samples and determine the effects of antibiotics, probiotics and autologous faecal microbiome transplantation (FMT) on the gut resistome in multiple cohorts. They demonstrated that a commercially available 11-strain probiotic blend of various strains of *Lactobacillus*, *Bifidobacterium*, *Streptococcus* and *Lactococcus* spp., can reduce the number of ARGs in colonization-permissive individuals, previously untreated with antibiotics. In contrast, after specified antibiotic treatment, the same probiotic mix intensified the antibiotic-mediated resistome expansion in the lower GI tract mucosa but did not seem to do so by directly transferring ARGs of their own. Specifically, the effect of probiotics on the stool resistome was restricted to the first day of supplementation as reflected in ARG-based β-diversity as well as to a transient increase in the number of observed ARGs. Contrarily, probiotics increased the differences in ARG-type configuration to the pre-supplementation baseline in endoscopic samples (6). Of note, the same group previously reported a subset of individuals who resist probiotic colonization in the GI tract mucosa, moreover even excluding these bacteria from the gut lumen (53, 54). It was sought to determine whether this influenced the effects of the tested probiotic mix on the resistome. It was found that the resistomes of permissive and resistant individuals were different at baseline, before receiving the probiotic. After probiotic supplementation, resistome was increasingly dissimilar to baseline in colonization-permissive but not in colonization-resistant individuals. This difference was associated with a reduction in ARG load and diversity. This led to the conclusion that possible probiotic-induced intestinal lumen reduction of ARG content in antibiotic-naïve individuals occurs in a colonization-dependent manner, and is therefore restricted to a subgroup of permissive individuals. Importantly, this effect could not be inferred from stool samples (6). Next, the resistome of adults with no known active infection who received a specific antibiotic combination (500 mg of oral ciprofloxacin two times per day and 500 mg of oral metronidazole three times per day) for 1 week was analysed from stool, provided prior and during the antibiotic treatment, as well as from colonoscopy samples, taken after treatment. Due to sample heterogeneity, the effects of antibiotic treatment on the ARG subtype diversity could not be conclusively shown in stool, suggesting that stool sampling should be considered insufficient when assessing the changes in gut resistome. However, lower GI tract endoscopic sampling enabled the detection of gut resistome expansion seen in both the number and variety of ARG content of individuals previously subjected to antibiotic treatment (6). Most significantly, when applied after antibiotic treatment, the supplemented probiotic mix was associated with increased ARG content and resistome expansion in endoscopic samples. However, the supplemented probiotic strains did not seem to be the direct source of these ARGs. Namely, genome assembly and ARG annotation of the probiotic blend and the gut resistome led to the conclusion that the majority of ARG types found in the probiotic belonged to the macrolide-lincosamide-streptogramin and tetracycline ARG type (*penA*, *lmrD*, *ermX*, *lmrP*, *lmrC*, *emeA*, *ileS*, and *tetW*), while only the vancomycin resistance gene, *vanG*, importantly undetected in the supplement, was significantly elevated in the post-antibiotic probiotic-treated individuals. Further analyses had shown that while probiotics inhibited the recovery of microbial diversity, they promoted the expansion of a number of commensal bacteria, four of which were significantly correlated with *vanG* abundance (*Clostridium citroniae*, *Clostridium leptum*, an unnamed *Blautia* sp., and *Romboutsia timonensis*). This gene has previously been demonstrated to horizontally transfer within the human gut (55, 56). This could potentially facilitate the horizontal transfer of ARGs, but whether this really occurs has yet to be determined. However, the conclusion that the probiotic-stimulated inhibition of microbiome recovery after antibiotic treatment allowed for the expansion of species that carry the clinically relevant ARGs could safely be reached (6). Therefore, it was suggested that the gut resistome changes may result from the ecological effects of probiotic supplements in the gut microbiome niche, rather than the direct HGT, which further highlights the importance of considering the ecological context in which probiotics are supplemented. Namely, as described above, when colonization is resisted by the host microbiome, probiotics do not elicit any benefits on the resistome. On the other hand, microbiome ablation by antibiotics supports probiotic colonization. Nevertheless, in this niche, probiotics pronouncedly inhibit most members of the microbiome except for the several strains that likely carry the ARGs, thus inducing resistome expansion. This effect was also shown to be ecologically conserved across host species, as similar strains and resistance genes appeared in humans and mice (*Blautia* spp. and vancomycin resistance genes) (6). While in the herein study the source of the enriched resistome was the microbiome itself rather than the supplemented probiotics, in previously mentioned data was presented the presence of ARGs in many commercially available probiotic foods and supplements, which may, therefore, be seen as a reservoir for resistome expansion in the gut. Thus, the possibility of direct horizontal transfer of resistance genes from probiotics to commensals and pathogens in the gut should not be neglected in further studies and scrutiny of probiotic safety. The extent to which personalized differences in probiotic colonization play a role in modulating their clinical efficacy is also yet to be determined.

It seems, however, that colonization permissiveness is in fact key to enabling any clinically significant effects of probiotics on the resistome. This is supported by the rapid recovery of the resistome from antibiotic-associated expansion after autologous FMT. Namely, if autologous, FMT offers greater compatibility between the host and transplanted microbiome and improves the likelihood of successful entrenchment. Persistent resistome disruption was observed more than 3 months after probiotic supplementation ceased, suggesting that the effects of probiotics on the gut resistome may be persistent and increasing the possibility that HGT events occurred within the gut microbiome (6). However, persistent post-antibiotics dysbiosis associated with probiotics may also contribute to ARG persistence by possibly reducing the fitness cost of ARG carrying, again rendering the perceived effects primarily ecological (57, 58). Notably, probiotics were supplemented after antibiotics and not concomitantly to disentangle the respective gut microbiome effects of probiotics and antibiotics (6). Thus, additional work is required to determine the effect of their concurrent administration on the gut resistome.

## Conclusion

Although limited by the scarcity of the presented studies, the reach of this overview may be deemed sufficient to suggest that wide and unregulated consummation of probiotic supplements and fermented foods and beverages could in time contribute to the global emergence of AMR and GI resistome expansion. The herein presented studies suggest that the dynamic unfolding of fermenting processes may increase the ARG content and variety in both artisan and commercial fermented products, despite the strict monitoring of the antibiotic use during animal husbandry and of the ARG content of standardized starter cultures. Furthermore, the presented data indicate that, in parallel to large-scale cohort studies oriented to the decryption and validation of the efficacy of probiotics, probiotic safety should also be monitored much more closely and intensely, particularly when concerning their concomitant use with antibiotics. To that end, additional work with greater variety and, if possible, concurrent administration of antibiotics and probiotics and longer follow-up periods is needed. More importantly, apart from the already intensified great-sequencing-depth genomic analyses of the probiotic ARG content and the potential for direct horizontal and vertical transfer of the underlying characteristics to commensal and pathogen GI species, the influence of probiotic products on the gut resistome should also be studied through the ecological lens. Namely, in future studies, it should seem beneficial that the GI tract would be viewed as a niche whose species and resistome content may be altered by probiotics only if the conditions for colonization are met, for instance when the specificities of an individual’s gut microbiome allow for this, or due to acquired dysbiosis, as seen after antibiotic use or in multiple diseases. The herein reviewed data suggest that in order to do this, thus far largely neglected *in situ* (endoscopic) sampling of the gut microbiome is required to fully evaluate the putative risk of ARG selection, transfer and expansion in both human and animal GI niches.

## Author contributions

MR, DK, and IG: the idea and conceptualisation. DK, MJ, IG, NO, and LR: review and editing. All authors writing the manuscript, read, and agreed to the final version of the manuscript.
